# Graphene-Assisted Polymer Waveguide Optically Controlled Switch Using First-Order Mode

**DOI:** 10.3390/polym13132117

**Published:** 2021-06-28

**Authors:** Yue Yang, Jiawen Lv, Baizhu Lin, Yue Cao, Yunji Yi, Daming Zhang

**Affiliations:** 1 State Key Laboratory of Integrated Optoelectronics, College of Electronic Science and Engineering, Jilin University, 2699 Qianjin Street, Changchun 130012, China; yangyue19@mails.jlu.edu.cn (Y.Y.); lvjw18@mails.jlu.edu.cn (J.L.); linbz17@mails.jlu.edu.cn (B.L.); yuecao17@mails.jlu.edu.cn (Y.C.); 2 College of New Materials and New Energies, Shenzhen Technology University, Shenzhen 518000, China

**Keywords:** optically controlled switch, graphene, photo-thermal effect

## Abstract

All-optical devices have a great potential in optical communication systems. As a new material, graphene has attracted great attention in the field of optics due to its unique properties. We propose a graphene-assisted polymer optically controlled thermo-optic switch, based on the E^x^_01_ mode, which can reduce the absorption loss of graphene. Graphene absorbs 980 nm pump light, and uses the heat generated by ohmic heating to switch on and off the signal light at 1550 nm. The simulation results show that, when the graphene is in the right position, we can obtain the power consumption of 9.5 mW, the propagation loss of 0.01 dB/cm, and the switching time of 127 μs (rise)/125 μs (fall). The switching time can be improved to 106 μs (rise) and 102 μs (fall) with silicon substrate. Compared with an all-fiber switch, our model has lower power consumption and lower propagation loss. The proposed switch is suitable for optically controlled fields with low loss and full polarization. Due to the low cost and easy integration of polymer materials, the device will play an important role in the fields of all-optical signal processing and silicon-based hybrid integrated photonic devices.

## 1. Introduction

With the rapid growth in demand for information in today’s society, a series of optical components has been proposed and produced. Optical signal control is crucial in the optical transmissions. Signal control can be realized by the manipulation of a metal electrode, in turn achieving thermal optical or electrical optical switching functions [1,2,3,4]. The optically controlled method is another way of realizing the switch function. An all-optical switch has important applications in the fields of signal processing and optical communication. In recent years, the all-optical switch has attracted a great deal of attention due to its fast switching speed and its lack of photoelectric conversion. To date, there have been many all-optical switches based on different principles and structures [5,6,7,8]. Among them, graphene-assisted all-optical switches have quickly become the primary source of all-optical switch research due to their advantages of fast response speeds and low power consumption.

As a two-dimensional (2D) material, graphene has many obvious electrical, optical, physical, and chemical properties. It has been extensively investigated in the field of opto-electronics, such as in optical modulators [9,10], photodetectors [11], polarizers [12], and saturable absorbers [13]. Graphene is also a promising material in the all-optical devices due to its photo-thermal properties [14,15]. In 2015, Gan et al. reported a graphene phase shifter in a Mach–Zehnder interferometer (MZI) that relied on ohmic heating to induce a thermo-optic effect; this device could achieve a nearly linear slope of 0.091 π/mW when pumped by 980 nm [16]. In 2020, Rang Chu et al. reported an all-optical phase shifter and switch based on a graphene decorated side-polished twin-core fiber (TCF) Michelson interferometer (MI). Relying on graphene’s photo-thermal effects, it could achieve a nearly linear slope of 0.0102 π/mW near the wavelength of 1550 nm and its switching times were 55.8 ms (rise) and 15.5 ms (fall) [17]. Graphene has also made important discoveries in modulators. In 2011, Liu Ming et al. integrated graphene with a silicon waveguide to produce light modulator; the authors demonstrated a modulation of the guided light at frequencies over 1 GHz, together with a broad operation spectrum that ranged from 1.35 to 1.6 μm under ambient conditions [18]. In 2013, Li Wei et al. proposed an ultrafast all-optical graphene modulator using micronano fiber. They used graphene’s Pauli blocking phenomenon to saturate the absorption of light, and then the signal light passed through the fiber during the saturation time of the switching light. Using an optical pump probe, the modulator was measured as having a response time of 2.2 ps, a modulation depth of 38%, and a modulation rate of 200 GHz [9]. However, these switches require a large driving energy, which causes a strong thermal effect, meaning the device cannot work efficiently and stably. This is a practical problem that has not yet been solved. Additionally, the thermal response time is slower by milliseconds. The reported devices are mainly based on inorganic material. 

Recently, polymer has been introduced into the photothermal all-optical field. Due to its excellent photo-thermal effects, the integration of polymer waveguide and graphene is more efficient. At the same time, it reduces the switching threshold for all-optical devices. In 2019, it was reported that graphene had been integrated as an electrode heater for a polymer MZI waveguide thermo-optic device. The power consumption of the fabricated device was 2.1 mW, with a rise time of 1.58 ms, and a fall time of 1.57 ms [19]. Zeshan Chang et al. reported an analysis of all-optical loss modulation in a graphene-buried waveguide, based on the Pauli blocking effect [20]. At the same time, they reported that graphene has a large absorption of the transverse-electric (TE) wave, but a small absorption of the transverse-magnetic (TM) wave [21]. Therefore, in order to solve the problem of high absorption in relation to the TE wave, Lv et al. proposed a thermo-optical switch using first-order mode to reduce the absorption of graphene; subsequently, the absorption loss was reduced to 0.06 dB/cm [22]. In this article, the single-mode TE wave with larger absorption was selected as the pump light, while the first-order TE wave with smaller absorption was selected as the signal light.

In this study, we employed graphene’s excellent optical properties and efficient thermal properties to accomplish an alternative method for an all-optical switch. In order to reduce the absorption loss of graphene, a polymer MZI thermo-optical switch based on the E^x^_01_ mode was proposed. We used the finite element method (FEM) for simulation analysis. Due to the advantages of its fast switching speed and its lack of need for photoelectric conversion, this device could play an important role in the fields of all-optical signal processing and silicon-based hybrid integrated photonic devices. 

## 2. Materials and Methods

### 2.1. Materials

Thermo-photo switch is achieved due to graphene absorption. The graphene effective permittivity *ε_g_* (=*n_g_*^2^) can be calculated from the conductivity σ: [23]

(1) $$\varepsilon_{g}\left(\omega \right)=1+\frac{i\sigma }{\omega \varepsilon_{0}d_{g}},$$

where *ω* is the angular frequency of the light, and *ε*_0_ is the vacuum permittivity. The graphene conductivity *σ* is affected by the temperature, graphene electronic relaxation time, optical angular frequency, and graphene Fermi level. When the number of graphene (SixCarbon Technology, Shenzhen, China) layers is 1, the parameters of graphene conductivity, including intraband conductivity and interband conductivity, can be calculated using the Kubo formalism:

(2) $$\sigma =\sigma_{intra}+\sigma_{inter},$$

(3) $$\sigma_{intra}=\frac{-ie^{2}}{\pi \hbar^{2}\left(\omega +i\tau^{-1}\right)}\int_{0}^{\infty }\xi \left[\frac{\partial f_{d}\left(\xi \right)}{\partial \xi }-\frac{\partial f_{d}\left(-\xi \right)}{\partial \xi }\right]d\xi ,$$

(4) σinter=ie2(ω+iτ−1)πħ2∫0∞fd(−ξ)−fd(ξ)(ω+iτ−1)2−4(ξħ)2dξ,

(5) $$f_{d}\left(\xi \right)={\left[e^{\left(\frac{\xi -\mu_{c}}{K_{B}T}\right)}+1\right]}^{-1},$$

where $f_{d}\left(\xi \right)$ is the Fermi–Dirac distribution function, *μ_c_* = 0.1 eV is the chemical potential (after the transfer of a single layer of graphene), ħ is the reduced Planck’s constant, *τ* = 5 × 10^−13^ s is the relaxation time, *K_B_* is Boltzmann constant, and *T* is temperature. The calculated refractive indices parallel to the graphene surface are 2.41 + 2.19i @980 nm and 2.98 + 2.79i @1550 nm, respectively.

Compared with inorganic materials, polymers have a higher thermo-optical coefficient and thus require lower power. In this paper, SU-8 was used as the core of the waveguide. SU-8 (Kayaku Advanced Materials, Inc., Westborough, MA, USA) is a polymer material with a thermo-optic coefficient of −1.8 × 10^−4^. The upper cladding adopted Polymethyl Methacrylate (PMMA) (Tokyo Chemical Industry, Tokyo, Japan), which has a low refractive index. The lower cladding was silica. The related material parameters of the switch are shown in Table 1.

**Figure 1:**
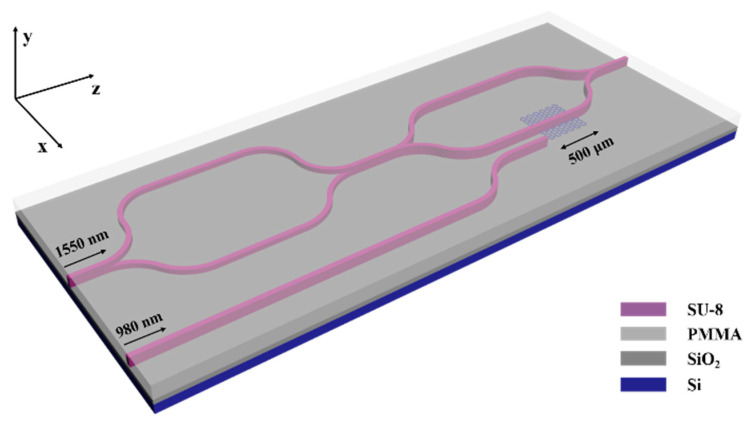
Schematic diagram of switch, including mode converter, pump light coupler, and first-order mode switch.

The device designed in this paper is composed of a mode converter and a MZI first-order mode optical switch, as shown in Figure 1. The all-optical switch is based on a balanced MZI, in which monolayer graphene is embedded in one of the arms. A vertical asymmetric mode converter is introduced at the input port to convert the E^x^_00_ mode into E^x^_01_ mode. The E^x^_01_ mode is output as a new signal light source to realize the thermo-optic modulation function of the symmetric MZI. We embedded monolayer graphene in the center of the waveguide with the same width as the core and a thickness of 0.34 nm. A first-order mode double-layer symmetry MZI optical switch can then be established with a graphene layer in one arm of the MZI branch to change the phase. A coupling waveguide was introduced on one arm of the MZI to couple the pump light at 980 nm. In this paper, two kinds of structures were used, one with silicon substrate and the other without substrate. The switch without substrate can be used in the flexible field. Compared to the switch without substrate, the switch with silicon substrate provides support for cross-section cutting and optical alignment, as well as faster heat dissipation, which can improve response speed.

### 2.2. Mode Converter

**Figure 2:**
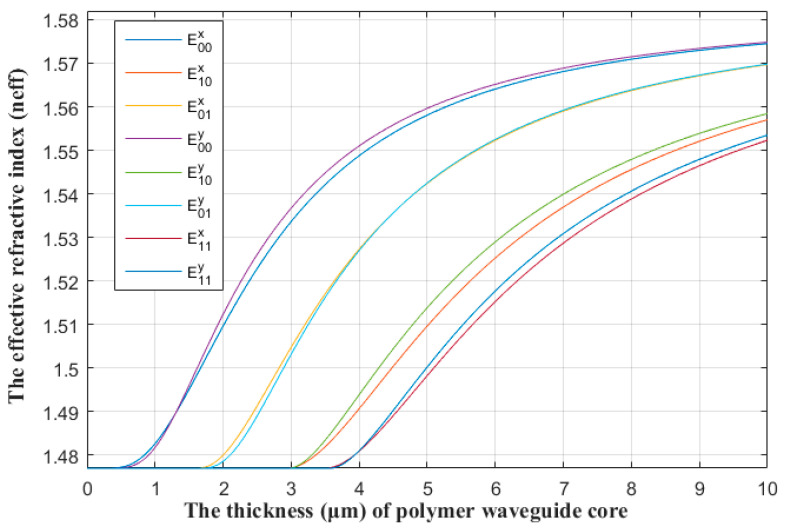
Relationship between the effective refractive index of different modes and the thickness of the polymer waveguide core.

To produce E^x^_01_ mode, we set the thickness of the waveguide at twice its width. For the signal light of 1550 nm, Matlab was used to calculate the relationship between the effective refractive index of different modes and the thickness of polymer waveguide core. The result is shown in Figure 2. It can be seen from the figure that when the thickness of the waveguide was 3 μm, the E^x^_01_ mode was satisfied. Therefore, the width of the waveguide was 1.5 μm and the thickness was 3 μm.

**Figure 3:**
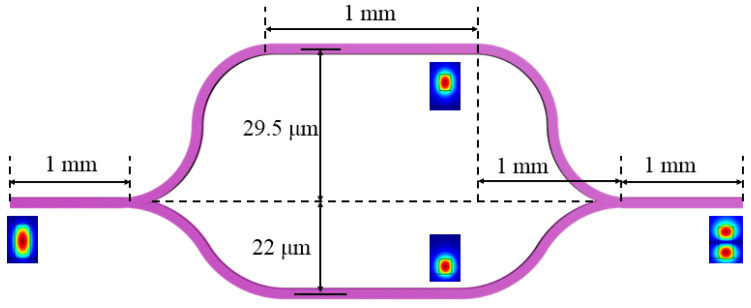
Schematic diagram of the mode converter.

**Figure 4:**
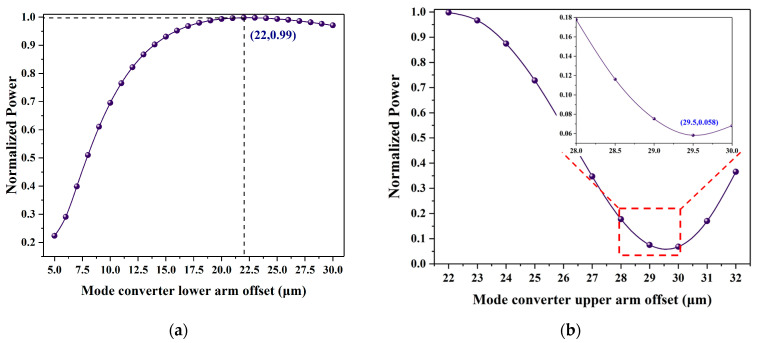
Scan results of the mode converter dimensions. (**a**) The normalized power as a function of mode converter lower arm offset; (**b**) the normalized power as a function of mode converter upper arm offset.

Most mode converters use long-period waveguide gratings or vertical directional coupling structures [24,25]. In this paper, a vertical asymmetric MZI structure was designed to be compatible with the switch fabrication process [22]. The schematic diagram of the mode converter is shown in Figure 3. It is composed of a vertical asymmetry MZI structure with two branches in different polymer waveguide layers. We used the BeamPROP method to scan and optimize the device’s parameters. Firstly, the offset of the symmetric bend waveguide was optimized, and the maximum output power was selected to ensure a small bending loss. Next, the offset of the upper arm waveguide was changed, and the minimum output power was selected to achieve the first-order mode conversion. Scanning results are shown in Figure 4. The length of the bend waveguide was determined to be 1000 μm, the lower arm offset was 22 μm, and the upper arm offset was 29.5 μm.

### 2.3. Coupler

The function of the coupler aims to avoid the waste of pump light caused by splitting the beam of the MZI structure. Coupling occurs along the transmission when the two waveguides are close and parallel. In this paper, the proportion of the input light coupled to another waveguide energy could be expressed by Equation (6),

(6) $$I\left(t\right)=\sin^{2}\left[\frac{\pi L_{d}\left(n_{0}-n_{1}\right)}{\lambda }\right],$$

where *n*_0_ and *n*_1_ are the effective refractive index of the single-order mode and the first-order mode, respectively, and *L_d_* is the length of the coupler. When the light couples into another waveguide, *I*(*t*) = 1, *L_d_* is an odd multiple of the coupling length; the coupling length becomes

(7) $$L=\frac{\lambda {\left(n_{0}-n_{1}\right)}^{-1}}{2}.$$

**Figure 5:**
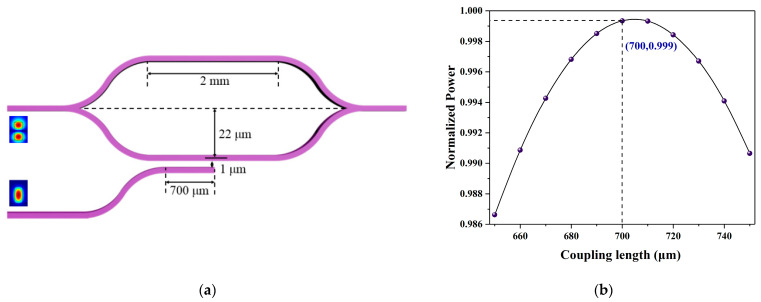
(**a**) Schematic diagram of coupler. (**b**) The normalized power as a function of the coupling length.

The structure of the coupler is shown in Figure 5a. The coupling length was scanned using BeamPROP at a coupling distance of 1 μm, as shown in Figure 5b. When the coupling length is 700 μm, the coupling efficiency is 99.9%.

### 2.4. Method

**Figure 6:**
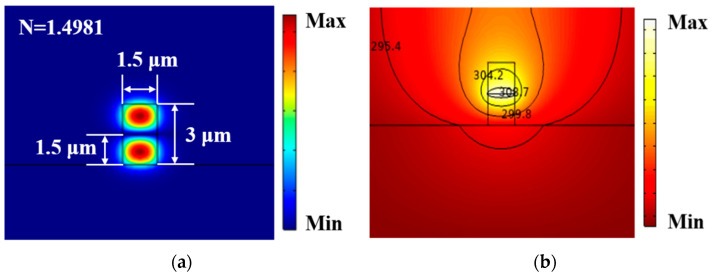
(**a**) Electric field diagram of the first-order mode; (**b**) schematic diagram of thermal field.

In this section, we discuss the research methods of an optically controlled switch. The COMSOL was used to calculate the electric field. First, a three-dimensional (3D) model was built to calculate the electric field generated by the pump at 980 nm. The grid was constructed as a sweep grid, and the effective refractive index was searched using boundary mode analysis, so as to calculate the single-mode at 980 nm. The absorption of graphene to the TE single-mode is particularly large, which limits the length of the modulation arm. Considering power consumption, the final length was selected at 500 μm. In this paper, a thermo-optic switch of MZI structure was used to heat an arm to change the effective refractive index of the waveguide. The phase difference between the two arms can be calculated by Equation (8),

(8) $$\Delta \varphi =\frac{2\pi }{\lambda }\Delta NL,$$

where Δφ is the waveguide phase change, λ is the optical wavelength, Δ*N* is the effective refractive index difference between the two arms, and *L* is the length of the heating electrode. The effective refractive index can be calculated by COMSOL 2D model and searched by mode analysis, so as to calculate the effective refractive index of the waveguide before and after heating. The electric field diagram of the first-order mode is shown in Figure 6a, with a propagation loss of 1.5 dB/cm. The effective refractive index change in the corresponding structure at different temperatures can be used when the phase change is π to calculate the temperature difference between the waveguide cores of the two arms. The required temperature difference was calculated to be 17.85 K. 

The thermal field can be calculated from the electric field. To simplify our simulation, as the length of graphene is much larger than the thickness of the waveguide, and the longitudinal temperature gradient along the waveguide resulting from the variation in graphene absorption is small, we omitted the longitudinal dependence of the temperature distribution. With continuous pumping, the temperature distribution in the waveguide T is described by the steady-state heat conduction equation [16]

(9) $$-k\nabla^{2}T=Q_{s},$$

where *k* is the thermal conductivity of the material and *Q_s_* is the heat source per unit volume generated by graphene absorption [26]

(10) $$Q_{s}=\frac{1}{2}\varepsilon_{0}\omega Im\left(\varepsilon_{r}\right){\left|E\right|}^{2},$$

where *ε*_0_ is the permittivity of vacuum, *ω* is the angular frequency of light, and *ε_r_* is the relative permittivity of graphene. The thermal field can therefore be calculated from the above formulas. We used graphene as a surface heat source in our simulation.

According to the principle of heat conduction, the thermal field distribution satisfies the Fourier heat conduction equation for homogeneous solid material. Only the steady-state case is considered as: $k\nabla^{2}T=0$, where k is the thermal conductivity of the material. When the homogeneous medium is in contact with other media, the heat transfer at the contact surface should be considered; in other words, this is the boundary problem. The usual heat transfer cases are thermal diffusion, thermal radiation and thermal convection. The first boundary condition is applied in this paper. As silica dissipates heat quickly, the lower surface of the substrate can be regarded as room temperature. On the left and right surfaces, the thermal conductivity is zero at a steady state as both sides are wide enough relative to the core. For the upper boundary of the waveguide, the polymer material is free convection heat transfer with the air, and $q=a\left(T-T_{\infty }\right)$, where q is the heat transfer under unit area, and a is the convective heat transfer coefficient. For undisturbed air, a = 5 W/(m^2^·k) and the heat field distribution of the waveguide can be obtained as shown in Figure 6b. 

When calculating the switching time, the transient equation is used to describe [27]

(11) $$\rho c\frac{\partial T}{\partial t}=\nabla \left(k\nabla T\right)+Q_{s},$$

where *T* is the unknown temperature as a function of time and space, *ρ* is the mass density of material, *c* is the heat capacity at constant pressure of material, and *k* is the thermal conductivity of the material.

## 3. Results and Discussion

**Figure 7:**
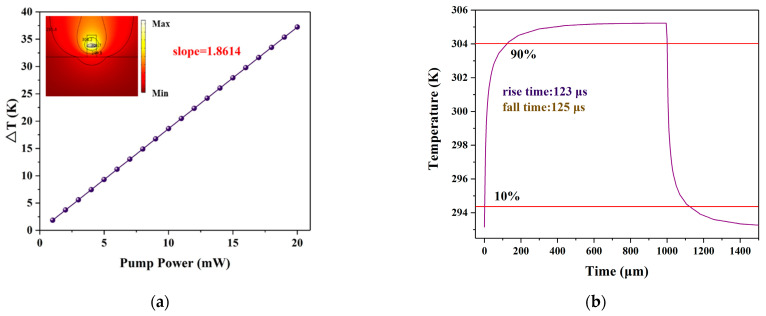
Switching characteristics without silicon substrate. (**a**) The relationship between waveguide temperature variation and pump power; (**b**) time response of optical switch.

The power consumption of the switch is our concern, so we calculated the relationship between waveguide temperature variation and pump power. The result shows a linear slope of nearly 1.8614 K/mW in Figure 7a. This means that a temperature change of 1.8614 K in the core can be realized with 1 mW of pump power. When the pump power is 9.6 mW, the phase difference between the two arms of the switch is π to realize switching off. The propagation loss is 1.4 dB/cm. The pump light at 980 nm was set as a square wave signal, and the transient response of the switch was calculated. The switching time obtained is shown in Figure 7b, with a rise time of 123 μs and fall time of 125 μs.

**Figure 8:**
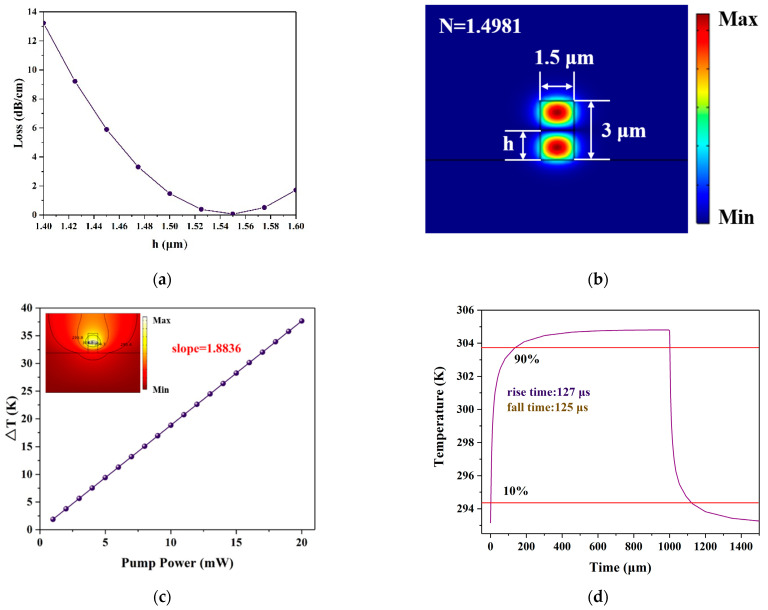
Switching characteristics without silicon substrate. (**a**) The relationship between the loss and graphene vertical position h; (**b**) electric field diagram of the first-order mode; (**c**) the relationship between waveguide temperature variation and pump power; and (**d**) time response of the optical switch.

Due to the differences in the upper and lower cladding materials for the waveguide structure used in this paper, the refractive index is different. As such, the smallest part of the first-order mode light field is not in the middle of the waveguide. The optimized result is shown in Figure 8a. When the vertical position of graphene was 1.55 μm, the propagation loss was 0.01 dB/cm. The switching performance of the optimized structure was simulated. The switching performance was analyzed by FEM. As shown in Figure 8, the required pump power is 9.5 mW, the propagation loss is 0.01 dB/cm, the rise time is 127 μs, and the fall time is 125 μs.

Comparing the two switching properties, we find that when the graphene is at 1.55 μm, the propagation loss and power consumption are reduced. As the pump power decreases, the switching time increases.

**Figure 9:**
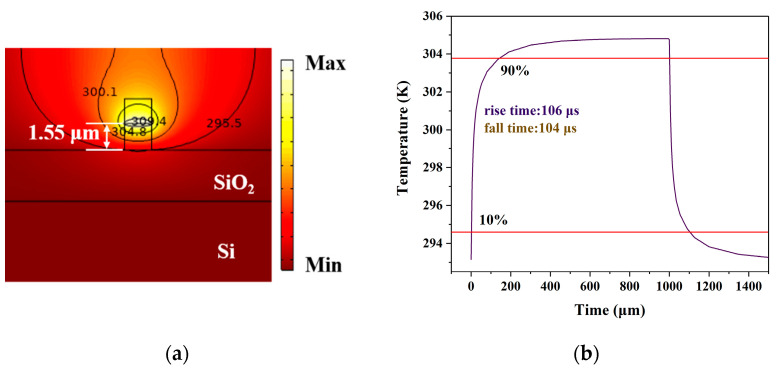
Switching characteristics with silicon substrate. (**a**) Thermal field diagram; (**b**) time response of the optical switch.

The above two switches did not have a silicon substrate. In order to improve switching time, a silicon substrate switch was proposed. For a waveguide with a thickness of 3 μm, when the vertical position of graphene was 1.55 μm, the propagation loss is 0.07 dB/cm, and the switching performance of the optimized structure was simulated. As shown in Figure 9, the required pump power is 10.3 mW, the propagation loss is 0.07 dB/cm, the rise time is 106 μs and the fall time is 102 μs. Compared with the switch without substrate, we find that the silicon accelerates the heat dissipation of the switch and improves switching time, however, power consumption and loss increase. 

Compared with the traditional optical waveguide switch, the optical switch proposed in this paper does not need photoelectric conversion, so the integration degree is improved; compared to all-fiber optical switch, our switch has lower power consumption and lower propagation loss. This device reduces the loss of TE mode and could play an important role in the field of all-optical signal processing and silicon-based hybrid integrated photonic devices.

## 4. Conclusions

In this article, we proposed a graphene-assisted polymer optically controlled thermo-optic switch based on E^x^_01_ mode. Graphene absorbed pump light at 980 nm to generate heat, which was used to modulate signal light at 1550 nm. The simulation results by FEM showed that the power consumption of the switch was 9.5 mW, the propagation loss was 0.01 dB/cm, the rise time of the switch was 127 μs, and the fall time was 125 μs. Switching times improved to 106 μs (rise) and 102 μs (fall) with the addition of silicon substrate. Compared to the traditional optical waveguide switch, the optical switch proposed in this paper does not need photoelectric conversion, thus, the degree of integration is improved; compared to all-optical switch of fiber, our switch has lower power consumption and a lower propagation loss. Due to the low cost and easy integration of polymer materials, this device will play an important role in the fields of all-optical signal processing and silicon-based hybrid integrated photonic devices.

## Figures and Tables

**Table 1:** Related material parameters in the switch.

Material	Refractive Index	Heat Capacity at Constant Pressure (J/(kg·K))	Thermal Conductivity (W/(m·K))	Density (kg/m^3^)
SU-8	1.588 @980 nm 1.582 @1550 nm	1200	0.2	1190
PMMA	1.48 @980 nm 1.477 @1550 nm	1420	0.19	1180
Silica	1.444 @980 nm 1.45 @1550 nm	730	1.4	2200
Graphene	2.41 + 2.19i @980 nm 2.98 + 2.79i @1550 nm	740	5300	1060

## References

[1] Elbialy S., Yousif B., Samra A. (2020). Modeling and Performance Enhancement of Active Hybrid Plasmonic Electro-optic Routing Switch. Plasmonics.

[2] Zhang X., Chen H., Liu L., Shang J., Yu L., Li K., Dong J., Qiu W., Guan H., Lu H. (2020). An Optical Switch Based on Electro-Optic Mode Deflection in Lithium Niobate Waveguide. IEEE Photonics Technol. Lett..

[3] Sun Y., Cao Y., Wang Q., Yi Y., Sun X., Wu Y., Wang F., Zhang D. (2018). Polymer thermal optical switch for a flexible photonic circuit. Appl. Opt..

[4] Cao Y., Yi Y.J., Lin B.Z., Sun Y., Che X.C., Zheng J., Wang F., Zhang D.M. (2019). Polymer/glass hybrid DC-MZI thermal optical switch for 3D-integrated chips. RSC Adv..

[5] Yang X., Long Q., Liu Z., Zhang Y., Yang J., Kong D., Yuan L., Oh K. (2019). Microfiber interferometer integrated with Au nanorods for an all-fiber phase shifter and switch. Opt. Lett..

[6] Wu L., Jiang X., Zhao J., Liang W., Li Z., Huang W., Lin Z., Wang Y., Zhang F., Lu S. (2018). MXene-Based Nonlinear Optical Information Converter for All-Optical Modulator and Switcher. Laser Photonics Rev..

[7] Wu K., Guo C., Wang H., Zhang X., Wang J., Chen J. (2017). All-optical phase shifter and switch near 1550nm using tungsten disulfide (WS2) deposited tapered fiber. Opt. Express.

[8] Wang C., Wang Y., Jiang X., Xu J., Huang W., Zhang F., Liu J., Yang F., Song Y., Ge Y. (2019). MXene Ti_3_C_2_T_x_: A Promising Photothermal Conversion Material and Application in All-Optical Modulation and All-Optical Information Loading. Adv. Opt. Mater..

[9] Li W., Chen B.G., Meng C., Fang W., Xiao Y., Li X.Y., Hu Z.F., Xu Y.X., Tong L.M., Wang H.Q. (2014). Ultrafast All-Optical Graphene Modulator. Nano Lett..

[10] Gao L., Ran H., Cao Y., Li Y., Huang W., Huang L., Feng D., Tang X., Zhu T. (2019). Coherent optical modulation of graphene based on coherent population oscillation. Opt. Lett..

[11] Grotevent M.J., Hail C.U., Yakunin S., Dirin D.N., Thodkar K., Barin G.B., Guyot-Sionnest P., Calame M., Poulikakos D., Kovalenko M.V. (2019). Nanoprinted Quantum Dot-Graphene Photodetectors. Adv. Opt. Mater..

[12] Cai K.S., Li Y.E., Wei W.J., Mu X.J., Ma A.N., Wang Z., Song D.M. (2018). TM-pass polarizer based on multilayer graphene polymer waveguide. Optoelectron. Lett..

[13] Zhang R.L., Wang J., Liao M.S., Li X., Guan P.W., Liu Y.Y., Zhou Y., Gao W.Q. (2019). Generation of wide-bandwidth pulse with graphene saturable absorber based on tapered fiber. Chin. Phys. B.

[14] Wu K., Wang Y., Qiu C., Chen J. (2018). Thermo-optic all-optical devices based on two-dimensional materials. Photonics Res..

[15] Wang Y., Gan X., Zhao C., Fang L., Mao D., Xu Y., Zhang F., Xi T., Ren L., Zhao J. (2016). All-optical control of microfiber resonator by graphene’s photothermal effect. Appl. Phys. Lett..

[16] Gan X., Zhao C., Wang Y., Mao D., Fang L., Han L., Zhao J. (2015). Graphene-assisted all-fiber phase shifter and switching. Optica.

[17] Chu R., Guan C., Bo Y., Liu J., Shi J., Yang J., Ye P., Li P., Yang J., Yuan L. (2020). Graphene decorated twin-core fiber Michelson interferometer for all-optical phase shifter and switch. Opt. Lett..

[18] Liu M., Yin X., Ulin-Avila E., Geng B., Zentgraf T., Ju L., Wang F., Zhang X. (2011). A graphene-based broadband optical modulator. Nature.

[19] Wang X., Jin W., Chang Z., Chiang K.S. (2019). Buried graphene electrode heater for a polymer waveguide thermo-optic device. Opt. Lett..

[20] Chang Z., Chiang K.S. (2019). All-optical loss modulation with graphene-buried polymer waveguides. Opt. Lett..

[21] Chang Z., Chiang K.S. (2016). Experimental verification of optical models of graphene with multimode slab waveguides. Opt. Lett..

[22] Lv J., Yang Y., Lin B., Cao Y., Zhang Y., Li S., Yi Y., Wang F., Zhang D. (2019). Graphene-embedded first-order mode polymer Mach-Zender interferometer thermo-optic switch with low power consumption. Opt. Lett..

[23] Cao Y., Lin B., Sun Y., Che X., Yi Y., Wang F., Zhang D. (2018). Thermal tuning of graphene-embedded waveguide filters based on the polymer-silica hybrid structure. RSC Adv..

[24] Wang W., Wu J., Chen K., Jin W., Chiang K.S. (2017). Ultra-broadband mode converters based on length-apodized long-period waveguide gratings. Opt. Express.

[25] He G., Gao Y., Xu Y., Ji L., Sun X., Wang X., Yi Y., Chen C., Wang F., Zhang D. (2018). Design and fabrication of three-dimensional polymer mode multiplexer based on asymmetric waveguide couplers. J. Opt..

[26] Chen X., Chen Y., Yan M. (2012). Nanosecond Photothermal Effects in Plasmonic Nanostructures. ACS Nano.

[27] Wan T., Guo Y., Tang B. (2019). Photothermal modeling and characterization of graphene plasmonic waveguides for optical interconnect. Opt. Express.

